# Efficient RFLP-based Protocol for Routine Authentication of Drosophila

**DOI:** 10.17912/micropub.biology.001949

**Published:** 2026-01-21

**Authors:** Melika Ghasemi Shiran, Nick P. Bailey, Lauren McCann, Natalia Rivera-Rincón, Emma Saurette, Laurie S. Stevison

**Affiliations:** 1 Biological Sciences, Auburn University, Auburn, Alabama, United States; 2 Centre for Biological Diversity, School of Biology, University of St Andrews, St Andrews, Scotland, United Kingdom; 3 Evolution, Ecology, and Organismal Biology, University of California, Riverside, Riverside, California, United States

## Abstract

Authentication of strains is important for preventing genetic contamination before any experiment, which can compromise reproducibility and lead to misleading results. Here, we developed an approach that combines computational single nucleotide polymorphism (SNP) identification with molecular validation using restriction fragment length polymorphisms (RFLPs). This workflow enables rapid and precise confirmation of strains in an inexpensive, reproducible, and easily adaptable way for long-term stock maintenance across laboratories. We apply this protocol to *Drosophila melanogaster* from the Drosophila Genome Resource Panel (DGRP), which are commonly used in fruit fly research, providing a reliable context for ensuring the integrity of Drosophila genetic resources.

**Figure 1. Overview of the workflow for strain identification and maintenance:**
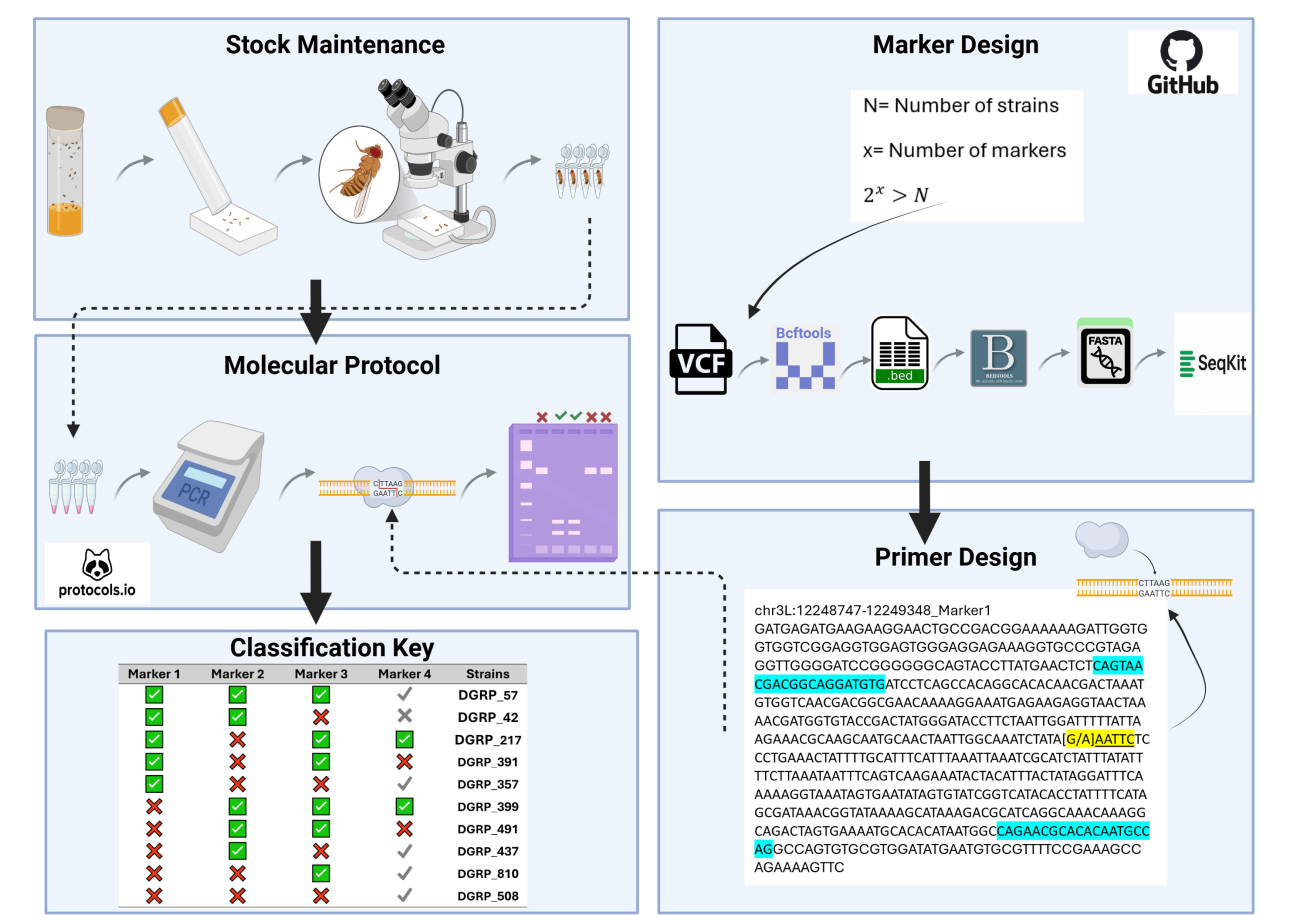
The workflow is divided into two complementary components. The right-hand side of the figure shows the one-time computational setup for marker selection and primer design. The primer design panel shows the forward and reverse primer binding regions (blue) and the polymorphic site [G/A] (yellow). The presence of G creates a restriction enzyme recognition site for EcoR1 which recognizes ‘GAATTC’, resulting in a cut. All computational scripts and resources are available on GitHub. The left-hand side shows the routine laboratory workflow, including stock maintenance and PCR amplification. The green check mark and red X symbols indicate the expected “cut” and “not cut” results. See the detailed Protocol for expected gel results. For Marker 4, gray checkmarks and X marks indicate that this marker is not necessary for differentiating those strains and can be excluded for efficiency in the molecular workflow. The molecular protocol is available on Protocols.io. Together, these steps allow consistent strain identification and routine verification of genetic strains. Created in BioRender. Shiran, M. (2025) https://BioRender.com/b5ycy6x

## Description

Reliable genetic maintenance of Drosophila strains is one of the most crucial steps in laboratories (Bangham, 2019; Ashburner et al., 2005). Genetic differences have been shown repeatedly to significantly influence experimental outcomes in gene function as well as behavioral traits (Ayroles et al., 2015; Chandler et al., 2014; Mackay & Huang, 2018; Saltz et al., 2017; Yanagawa et al., 2020). As a result, misidentified or genetically contaminated fly lines can result in unreproducible results, complicating the assessment of any experimental result.

Established authentication techniques, including Sanger sequencing or whole-genome sequencing of individual fly strains, have been helpful for mutation discovery but are often costly and time-consuming for routine quality control (Blumenstiel et al., 2009; Gerhold et al., 2011). Stock centers provide best practice guidelines for preventing contamination, such as mites, fungi, and mold (Bangham, 2019; Hales et al., 2015), but they do not provide standardized guidelines for preventing possible sources of genetic contamination. Visual authentication may be sufficient for mutant strains, but wild-type strains currently lack a standard molecular protocol. Due&nbsp;to the rapid generation time of Drosophila, a&nbsp;fast&nbsp;and&nbsp;inexpensive&nbsp;approach is necessary. Here, we present an integrated computational and molecular laboratory approach for the authentication of commonly used Drosophila strains (Mackay et al., 2012). In the computational step, single nucleotide polymorphisms (SNPs) that vary among sequenced wild-type stocks and overlap with restriction enzyme recognition sites are identified based on publicly available genome datasets (Mackay et al., 2012). For *D. melanogaster* stocks, the resulting gel patterns reveal whether the stock matches the expected strain or has been mislabeled or contaminated. Restriction fragment length polymorphisms are classically used to distinguish strains (Botstein et al., 1980). In the wet lab step, the applicable genomic areas are amplified by PCR, and subsequently, digested via restriction enzyme, and visualized via gel electrophoresis.&nbsp;The expected fragment sizes on the gel electrophoresis indicate the successful authentication of each Drosophila stock shown in Figure 1.

Ultimately, stock authentication should be the initial step of any Drosophila research workflow, like other model organisms, to ensure the reproducibility, accessibility, and adaptability of experimental outcomes across laboratories (Yoshiki et al., 2022).

## Methods

This protocol consists of two steps: 1) computational marker design and 2) molecular validation. We apply this protocol to *Drosophila melanogaster* strains. The computational pipeline is documented in detail on GitHub (https://github.com/StevisonLab/Drosophila-Genotyping; DOI: https://doi.org/10.5281/zenodo.18303467), and the molecular pipeline is documented in detail via Protocol.io (DOI: dx.doi.org/10.17504/protocols.io.q26g7n3mklwz/v1), which together enable validation of polymorphic markers suitable for distinguishing closely related strains. An overview of these steps is provided in Figure 1.

First, similar to how dichotomous species keys work, we used a power-of-two approach to determine how many genetic markers were needed to confidently identify each DGRP strain (Sokal & Rohlf, 2009, p. 46). In this context, each marker corresponds to whether a restriction site is cut or not cut. This logic ensures that each marker provides a binary piece of distinguishing information, enabling the unique identification of multiple unique strains using the smallest possible number of genetic markers. For example, by using only 8 markers, it is possible to distinguish up to 256 strains, exceeding the number of 205 available DGRP strains (Mackay et al., 2012). In this protocol, 10 DGRP strains were selected based on the strains that were in use in our lab at the time of protocol development. The smallest power of two greater than or equal to 10 is 16, corresponding to 2 ^4^ , so the four markers used here were sufficient to uniquely distinguish among these 10 strains. Each strain was identified by its specific combination of restriction cut patterns at these marker sites. This approach, along with the associated code, allows for the generation and distinction of additional strains. It is worth noting that once a set of markers is designed for a set of strains, adding additional strains may not lead to unique cut patterns. For example, in this protocol, the first three markers provided a maximum of 2 ^3^ =8 unique restriction patterns, which was insufficient to uniquely identify all selected strains. As a result, two pairs of DGRP lines shared identical cut patterns across markers 1–3 (Figure 1). To resolve this ambiguity, a fourth marker was added, which provided additional binary information and allowed these previously indistinguishable strain pairs to be uniquely identified. Importantly, the inclusion of this additional marker not only resolved the current overlap but also increased the total number of distinguishable strain patterns, allowing for the identification of additional strains if needed in future experiments.

Marker design used custom publicly available bash scripts, which are compatible between different Unix-based shells. Specifically, variant call format (VCF) files downloaded from the DGRP website were used to obtain markers that differentiate different DGRP strains. First, ‘bcftools’ was used to process the VCF file and filter variants that distinguish subsets of strains, until each line was individually identifiable (Danecek et al., 2021). The resulting candidate sites were converted to BED files, with 300 bp of flanking sequence for each variant. Next, the corresponding genomic sequences were extracted from the reference FASTA file by using ‘bedtools’ (Quinlan & Hall, 2010). The restriction enzyme EcoRI recognition sequence "GAATTC" was searched in these sequences to identify candidate markers at the variant location. It is worth noting that EcoRI was selected because it is easy to use, highly reliable, and inexpensive. In principle, this enzyme could easily be replaced based on experimental preference or availability, as long as there are sufficient restriction sites across the genome to maximize marker selection to resolve the strains of choice. Finally, ‘seqkit’ was used to calculate and rank sequences by GC content to aid in primer design (Shen et al., 2016). This combination of tools allowed the production of robust markers that could reliably distinguish each strain. It is worth noting that there are also stand-alone tools recently developed that could be used to generate these type of RFLP markers from a VCF file in a single step (Wesołowski et al., 2021).

Primer design was done using primer3 for each marker region identified (Untergasser et al., 2012). For DGRP markers, the forward primer Tm and GC content respectively ranged from 59.02 to 60.11°C, and 36% to 55%. Reverse primers ranged from 59.97 to 60.88°C, and 50% and 60%. The expected product size from PCR amplification was 326 to 433bp. Also fragment sizes for cut alleles DGRP Marker 1: 183bp + 250bp; Marker 2: 242bp + 146bp; Marker 3: 213bp + 113bp; Marker 4: 177bp +227bp. To confirm that each primer set specifically amplified the intended target and correctly distinguished alleles among the tested strains, we performed molecular validation using at least six independent fly replicates per line, each derived from at least two samples each from female and males, with consistent restriction cut patterns observed across independent experiments. Males were initially tested but later excluded because results were inconsistent, likely due to lower DNA yield from their smaller body size. For all subsequent experiments, only females were used to ensure consistent DNA quality. For all markers, the observed digestion patterns matched those predicted from the genome sequences. This validation process was also repeated for a set of wild-type *D. pseudoobscura* strains (DPSE, not shown) and is documented in detail in the molecular protocol available on Protocols.io. One initial DPSE marker set failed due to a lack of SNP confirmation in the strain and was replaced with an alternative.

The molecular pipeline, after confirmation, started with sample collection. Two replicate female flies from each strain shown in Figure 1 *of D. melanogaster* were anesthetized using CO₂, placed individually into wells of a 8-strip tube, and frozen. For the second step, a mixture of a squishing buffer and Proteinase K (Sigma P-6556) was used for DNA extraction (Gloor & Engles, 1992). Then, it was incubated at 37°C for 30 minutes, then at 95°C for 2 minutes, and frozen. Thirdly, PCR amplification was performed using GoTaq Green Master Mix with stock-specific primers with four markers for *D. melanogaster* ( *Promega* , 2021). &nbsp;The thermocycler program was set to an initial annealing of 60°C and then a standard touchdown method to amplify DNA exponentially. Fourth, restriction enzyme digestion was performed using EcoRI (R0101S) ( *NEB* , 2018). Reactions were incubated at 37°C for 60 minutes. Finally, gel electrophoresis with 2% agarose was captured with an Azure system under ethidium bromide settings. This protocol is repeated regularly (every 4-6 weeks) to ensure authentication of strains throughout their use in laboratory experiments.

## Reagents

**1. Fly Strains:**

**Table:** 

Strain	Species	Resource Reference ID (RRID)	Available From
*DGRP-42*	*Drosophila melanogaster*	BDSC_25193	Bloomington Drosophila Stock Center
*DGRP-57*		BDSC_29652	
*DGRP-217*		BDSC_28154	
*DGRP-357*		BDSC_25184	
*DGRP-391*		BDSC_25191	
*DGRP-399*		BDSC_25192	
*DGRP-437*		BDSC_25194	
*DGRP-491*		BDSC_28202	
*DGRP-508*		BDSC_28205	
*DGRP-810*		BDSC_28239	

**2. Primers:**

**Table:** 

Marker	Forward Primer	Reverse Primer	chr	start	end	SNP	Which strains are cut?
1	CAGTAACGACGGCAGGATGT	CTGGCATTGTGTGCGTTCTG	3L	12248747	12249348	A/G	57,42,217,391,357
2	ATGTATCGAGAGCACGGCAA	TTTTCACGGCGTTCTTTGGA	3L	1283464	1284065&nbsp;	C/T	57,42,399,491,437
3	TGCATACATTTATCCAAATCGCAAC	GCGTCAACAAGACCCACAAC&nbsp;	X	19069885	19070486&nbsp;	C/A	57,217,391,399,491,810
4	TCCCTTGGCTGCATTTGTCT	CATTTCGATCGCTCCCCCAG	2L	969206	969807	C/T	217,399*

*Note, because 2 ^3^ can distinguish up to 8 strains, the fourth marker is only required for the final two pairs of lines for authentication.

**3. Reagents and Equipment:**

·&nbsp;&nbsp;&nbsp;&nbsp;&nbsp; **1M Tris-HCl: Bis-tris/ Hydrochloric acid, buffer, 1M, Rigaku:** Rigaku Reagents 101443-554

·&nbsp;&nbsp;&nbsp;&nbsp;&nbsp; **EDTA: VWR® EDTA 0.5M, Biotechnology Grade:** VWR 97062-654

·&nbsp;&nbsp;&nbsp;&nbsp;&nbsp; **5M NaCl: Sodium chloride solution 5 M, sterile:** G-Biosciences 82023-090

·&nbsp;&nbsp;&nbsp;&nbsp;&nbsp; **Proteinase-K Solution:** Promega Corporation&nbsp;PAV3021

·&nbsp;&nbsp;&nbsp;&nbsp;&nbsp; **Promega&nbsp;GoTaq G2 Green Master Mix:** Promega Corporation&nbsp;M7822

·&nbsp;&nbsp;&nbsp;&nbsp;&nbsp; **Nuclease Free Water:** VWR 7732-18-5

·&nbsp;&nbsp;&nbsp;&nbsp;&nbsp; **10x Buffer EcoR1/SspI:** New England Bio Labs 76486-068

·&nbsp;&nbsp;&nbsp;&nbsp;&nbsp; **ECOR1:** New England Bio Labs 101641-106

·&nbsp;&nbsp;&nbsp;&nbsp;&nbsp; **Gel Loading Dye, Purple (6x):** New England Bio Labs 102877-816

·&nbsp;&nbsp;&nbsp;&nbsp;&nbsp; **Agarose Gel:** RPI 76344-692

·&nbsp;&nbsp;&nbsp;&nbsp;&nbsp; **TAE Buffer:** Promega Corporation V4271

·&nbsp;&nbsp;&nbsp;&nbsp;&nbsp; **Ethidium Bromide:** VWR 97064-602

·&nbsp;&nbsp;&nbsp;&nbsp;&nbsp; **Azure Gel Imager:** Azure Imaging Systems AZI200-01 - AZI600-01
